# Associations between perceived stress, sleep quality, and suicidal ideation among Chinese graduating-grade secondary school students: a multi-method study of the moderating role of physical exercise

**DOI:** 10.3389/fpubh.2026.1934268

**Published:** 2026-09-21

**Authors:** Tian Yin, Yanfeng Peng, Longjiang Chen, Xiaoying Lin, Feiqing Liang

**Affiliations:** 1 School of Physical Education, Yunnan Minzu University, Kunming, Yunnan, China; 2 School of Physical Education, Yunnan Normal University, Kunming, Yunnan, China; 3 The Department of Pharmacy, The Second People's Hospital of Longgang District, Shenzhen, China

**Keywords:** academic stress, adolescent mental health, high school students, machine learning, mediation, moderation, network analysis, perceived stress

## Abstract

**Objective:**

This study aimed to investigate the associations among perceived stress, sleep quality, physical exercise, and suicidal ideation among Chinese graduating-grade secondary school students. Specifically, this study examined whether sleep quality mediated the association between perceived stress and suicidal ideation and whether physical exercise moderated these relationships. In addition, machine-learning prediction models and network analysis were applied to identify important predictors and interconnected symptoms associated with suicidal ideation.

**Methods:**

A cross-sectional survey was conducted among ninth- and twelfth-grade students from 13 secondary schools in Yunnan Province, China. A total of 4,372 valid questionnaires were collected. Participants completed measures of perceived stress, sleep quality, suicidal ideation, and physical exercise. Mediation and moderated mediation analyses were conducted using PROCESS models after adjusting for demographic covariates. Furthermore, ridge regression and gradient boosting tree models were used to predict suicidal ideation, with Shapley additive explanations (SHAP) applied to evaluate predictor contributions. Network analysis was conducted to explore central and bridge nodes among study variables.

**Results:**

Perceived stress was positively associated with suicidal ideation, and sleep quality partially mediated this association. Physical exercise significantly moderated the direct association between perceived stress and suicidal ideation as well as the association between sleep quality problems and suicidal ideation. Specifically, higher levels of physical exercise weakened the positive relationships between perceived stress, sleep problems, and suicidal ideation. Machine-learning analyses showed that sleep quality problems, perceived stress, physical exercise, and their interaction terms contributed substantially to the prediction of suicidal ideation, with both models achieving an R^2^ of approximately 0.41 in the hold-out test set. Network analysis further identified subjective sleep quality, sleep latency, sleep disturbances, stress-coping burden, and physical exercise frequency as highly connected nodes within the network.

**Conclusion:**

Among Chinese graduating-grade secondary school students, perceived stress, poor sleep quality, and suicidal ideation were closely interconnected, whereas physical exercise appeared to attenuate these risk associations. Integrating stress management, sleep improvement strategies, and regular physical exercise into school-based mental health programs may provide potential approaches for suicide risk prevention among adolescents.

## 1 Introduction

Suicide is a major global public health concern. The World Health Organization has reported that more than 720,000 people die by suicide each year, making suicide the third leading cause of death among individuals aged 15–29 worldwide (1). Suicide is conceptualized as a continuum process, in which suicidal ideation represents the initial stage. Suicidal ideation refers to thoughts, desires, or plans to end one’s own life (2, 3). Epidemiological evidence indicates that suicidal ideation is relatively prevalent among children and adolescents, and those who experience suicidal ideation are at a substantially higher risk of subsequent suicide-related outcomes (4). In secondary school students, suicidal ideation is not merely a transient or isolated psychological phenomenon; rather, it may serve as an important risk signal for subsequent mental health problems, suicidal behaviors, and impaired social functioning (5, 6). Longitudinal studies have further demonstrated that adolescent suicidal ideation is closely associated with later psychopathology, suicide-related behaviors, and long-term social dysfunction (7). Therefore, identifying the factors associated with suicidal ideation in secondary school students, as well as elucidating its underlying mechanisms, is of critical importance for school-based mental health education and crisis intervention.

Among secondary school students, those in their final year experience multiple academic and developmental stressors, including entrance examinations, academic competition, parental expectations, and uncertainty about future trajectories. Prolonged stress may increase SI by affecting emotional regulation, recovery processes, and daily behaviors. Sleep quality represents a potential pathway linking stress to mental health outcomes, as sleep problems may impair emotional regulation and stress recovery capacity.

SI is a complex outcome arising from interactions among stress, sleep problems, health behaviors, and psychological adaptation. Traditional models can examine overall pathways but provide limited information about the relative importance of specific variables. Network analysis and predictive models may therefore provide complementary evidence by identifying key nodes and predictive contributors associated with SI.

### 1.1 Perceived stress and suicidal ideation

Perceived stress refers to an individual’s appraisal of the extent to which situations in life are unpredictable, uncontrollable, and overloading (8). Compared with objective stressful events, PS emphasizes individuals’ cognitive appraisal of environmental demands and their perceived capacity to cope with those demands (9).

Among final-year secondary school students, multiple stressors—such as entrance examinations, academic competition, performance evaluation, parental expectations, peer comparison, and uncertainty regarding future development—do not exert uniform effects on mental health. Instead, their impact largely depends on whether students perceive these demands as exceeding their coping capacity and controllability (10). When students persistently appraise stressors as unpredictable, uncontrollable, or unmanageable, their psychological resources may become chronically depleted, leading to heightened tension, anxiety, depressive symptoms, helplessness, and self-devaluation. From a theoretical perspective, PS may represent a key risk factor for SI (11). According to the stress-vulnerability framework, individuals exposed to sustained high levels of stress, particularly in the absence of sufficient psychological resources, are more likely to develop maladaptive cognitive appraisals and affective dysregulation (12, 13). For final-year secondary school students, elevated PS not only intensifies threatening interpretations of examination failure, academic fluctuations, and future uncertainty, but may also undermine problem-solving abilities, emotional regulation capacity, and hopefulness (14, 15). When students continuously perceive their current situation as unchangeable and future outcomes as uncontrollable, feelings of hopelessness, perceived failure, and diminished life meaning may emerge, thereby increasing the likelihood of SI (16). Accordingly, PS is not only a salient indicator of psychological risk in this population but may also serve as a key psychosocial determinant of variability in SI. Based on this rationale, the following hypothesis is proposed:

*H1*: PS is significantly positively associated with SI.

### 1.2 The mediating role of sleep quality

Sleep quality refers to an individual’s overall evaluation of their sleep experience, typically encompassing subjective sleep perception, sleep onset latency, sleep duration, sleep efficiency, sleep disturbances, and daytime functional status (17). Among final-year secondary school students, elevated perceived stress is generally associated with a sustained state of tension, worry, and heightened psychological burden. Such persistent stress exposure can disrupt normal sleep processes, leading to difficulties in sleep initiation, impaired sleep maintenance, reduced restorative sleep, and increased daytime fatigue (18). From the perspective of emotion regulation theory, sleep plays a central role in maintaining functional balance within the prefrontal–limbic system (19). Sleep deprivation is associated with reduced cognitive reappraisal capacity and impaired inhibitory control, while simultaneously increasing emotional reactivity to negative stimuli. These changes may facilitate the development of maladaptive interpretative biases and helpless cognitions, thereby elevating the risk of suicidal ideation (19). In addition, from a stress recovery framework, poor sleep quality compromises an individual’s ability to recover from daily stressors, making academic setbacks, interpersonal conflicts, and other stress-related events more likely to accumulate into sustained negative affective experiences (20, 21). Within this process, sleep quality may function as a mediating mechanism linking PS to SI, reflecting a statistical pathway through which stress experiences are translated into emotional distress and suicide-related cognitions. Therefore, PSQI may serve as an important bridging mechanism in the association between PS and SI.

For final-year students, high-stakes academic demands may lead to repetitive pre-sleep cognitive activity and impaired recovery. Poor sleep may further increase emotional difficulties and reduce coping capacity, suggesting that sleep quality may represent an important statistical pathway linking PS and SI.

*H2*: PSQI mediates the relationship between PS and SI.

### 1.3 Physical exercise as a moderator of the association between perceived stress and suicidal ideation

Physical exercise is typically defined as a subset of physical activity that is planned, structured, repetitive, and aimed at improving or maintaining physical fitness (22). More broadly, it represents a form of physical activity characterized by bodily movement produced by skeletal muscles that results in energy expenditure (23). Regular PE not only enhances physical functioning but also reduces the risk of mental health problems in adolescents through multiple pathways, including neurophysiological regulation, sleep improvement, stress relief, increased positive affect, and enhanced psychological resilience (24, 25). For final-year secondary school students, PE may provide an effective outlet for stress release and emotional regulation outside of academic demands. As such, even in the context of elevated perceived stress and sleep-related difficulties, engagement in regular PE may help maintain relatively stable psychological functioning.

From a stress-buffering perspective, PE may reduce the adverse effects of stress exposure on psychological outcomes. It may improve mood, enhance self-efficacy, promote social connection, and strengthen emotional regulation capacity, thereby weakening the translation of stress into SI.

For students facing intensive academic pressure, maintaining regular PE may provide additional coping resources and reduce vulnerability to negative self-evaluation and suicidal thoughts.

Accordingly, PE may serve as a key protective condition that weakens the positive association between PS and SI. Based on this rationale, the following hypothesis is proposed:

*H3*: PE moderates the relationship between PS and SI.

### 1.4 Physical exercise as a moderator of the association between sleep quality and suicidal ideation

In addition to moderating the relationship between PS and SI, PE may also moderate the association between sleep quality and SI. Specifically, when students experience poor PSQI, their emotional regulation capacity, cognitive control, and stress recovery abilities may be impaired, thereby increasing vulnerability to SI (19). From a stress-buffering perspective, PE, as an important modifiable behavioral resource, may attenuate the adverse effects of sleep problems on mental health outcomes (26). On the one hand, regular PE contributes to the improvement of sleep rhythms and sleep restoration processes. On the other hand, it enhances positive affect, self-efficacy, and psychological resilience, thereby weakening the negative impact of poor PSQI on SI (27–29). Furthermore, from the perspective of Conservation of Resources theory, sleep deprivation leads to continuous depletion of psychological and physiological resources, whereas PE may help replenish such resources by enhancing physical fitness and recovery capacity, thereby buffering the adverse psychological consequences of resource loss (30, 31). In addition, from the Interpersonal Theory of Suicide, thwarted belongingness and perceived burdensomeness are key mechanisms underlying SI. PE may reduce SI risk by enhancing social connectedness and promoting more positive self-evaluations, thereby further attenuating the impact of poor PSQI on SI (32, 33).

For final-year secondary school students, this relationship has important practical implications. Under conditions of intensive exam preparation and prolonged academic stress, sleep problems are highly prevalent. When students also lack regular PE, fatigue, emotional dysregulation, and negative cognitive processing resulting from insufficient or poor-quality sleep may further accumulate, thereby increasing the risk of SI (34). In contrast, higher levels of PE may provide additional psychophysiological recovery resources, thereby attenuating the positive association between PSQI problems and SI. Based on this rationale, the following hypothesis is proposed:

*H4*: PE moderates the relationship between PSQI and SI.

### 1.5 Current study

In summary, graduating-grade secondary school students are exposed to a concentrated period of educational transition and academic competition, during which perceived stress, sleep quality, physical exercise, and suicidal ideation may be closely interrelated. Elevated PS may increase the risk of SI, whereas impaired PSQI may represent an important mechanism through which stressful experiences are translated into suicidal thoughts (35). In parallel, PE, as a modifiable health behavior, may buffer the adverse association between sleep problems and SI (36). However, most existing studies have focused on single pathways or overall associations among variables. Relatively few have simultaneously examined stress-related risk transmission, the mediating role of PSQI, the moderating effect of PE, the predictive contribution of key variables, and the network structure linking these constructs among graduating-grade secondary school students. Therefore, the present study constructed a moderated mediation model and integrated machine-learning prediction with network analysis to investigate the correlates of SI among Chinese graduating-grade secondary school students from three complementary perspectives: explanatory pathways, predictive contribution, and node-level network structure (Figure 1).

**Figure 1:**
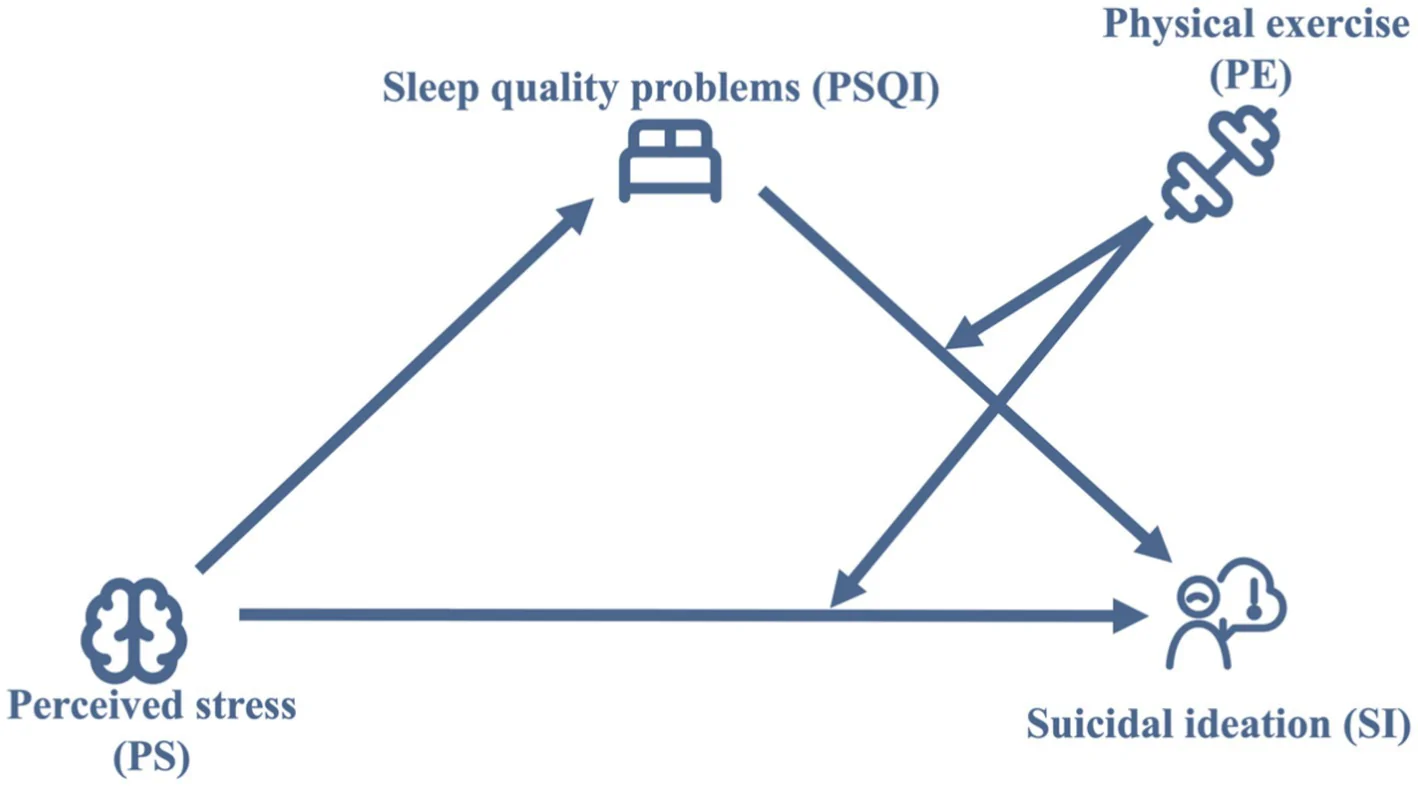
Moderated mediation model.

## 2 Methods

### 2.1 Participants

A school-level convenience sampling strategy combined with class-level cluster sampling was used to conduct an in-person questionnaire survey across 13 schools in Yunnan Province, China, between March and May 2026. At the school level, the participating schools were recruited non-probabilistically on the basis of accessibility, administrative feasibility, and willingness to participate. School recruitment was not based on a probability sampling frame, and no stratification or quotas were applied according to school type, gender composition, or boarding status. Within each participating school, intact Grade 9 and/or Grade 12 classes served as the sampling units, and eligible students in the selected classes were invited to participate. The participants were Grade 9 and Grade 12 students at critical educational transition points. Grade 9 students were preparing for the transition from junior to senior secondary education, whereas Grade 12 students were approaching the transition from senior secondary to higher education. Both groups therefore represented graduating-grade cohorts experiencing relatively high academic demands. Questionnaires were administered collectively on a class-by-class basis by uniformly trained research staff following standardized procedures to ensure consistency in questionnaire distribution, on-site administration, and data collection.

Before data collection, the research team informed school administrators, homeroom teachers, parents or legal guardians, and students about the study objectives, anonymity procedures, data-confidentiality measures, and the potentially sensitive mental health content of the questionnaire. As all participants were minors, informed consent was obtained from their parents or legal guardians through the participating schools, and the students provided voluntary assent before participation. Participants were informed that they could decline to answer any question or withdraw from the study at any time without adverse consequences. The questionnaires were completed anonymously and collected no names, national identification numbers, telephone numbers, or other directly identifying information. All data were used exclusively for aggregate-level statistical analyses. Given the inclusion of sensitive questions concerning suicidal ideation, all participants received the same mental health support information card listing school-based counseling services, local mental health services, the national mental health assistance hotline, and emergency support options. School counselors or appropriately qualified mental health professionals were available on site throughout data collection. If a student exhibited evident emotional distress, requested assistance, or directly disclosed a current risk of self-harm or suicide, questionnaire completion was immediately discontinued, and the student was referred confidentially to an on-site professional for further safety assessment. When necessary, the professional contacted the student’s parent or legal guardian, a specialist mental health service, or emergency services in accordance with a prespecified protocol approved by the ethics committee. Because the questionnaires were fully anonymous, the research team could not identify or trace individual participants based on their PANSI scores. Accordingly, universal access to mental health support resources and on-site professional assistance were provided to mitigate potential risks while preserving participants’ privacy. Ethical approval was obtained from the Ethics Committee of Yunnan Minzu University before data collection (Approval No. 2026021). The inclusion criteria were as follows: (1) current enrollment as a secondary school student; (2) enrollment in Grade 9 or Grade 12; (3) sufficient reading comprehension to complete the questionnaire independently; and (4) parental or guardian informed consent and the student’s voluntary assent. The exclusion criteria were: (1) failure to provide the required consent or assent; (2) evidently incomplete questionnaire responses; (3) substantial missing data on key variables; and (4) clearly patterned or internally inconsistent responses. A total of 5,301 questionnaires were distributed. After accounting for nonreturned questionnaires and excluding responses that did not meet the prespecified data-quality criteria, 4,372 valid questionnaires were included, yielding a valid response rate of 82.48%. Participant characteristics are presented in Table 1.

**Table 1:** Demographic characteristics and descriptive statistics of the main study variables.

Variable	Category	*N* (%)/ M ± SD
Age	M ± SD	16.07 ± 1.52
Gender	1 = Male	2,705 (61.9)
	2 = Female	1,667 (38.1)
Grade	1 = Grade 9	2,136 (48.9)
	2 = Grade 12	2,236 (51.1)
Only child	1 = Yes	1797 (41.1)
	2 = No	2,575 (58.9)
Boarding status	1 = Boarding	3,059 (70.0)
	2 = Commuting	1,313 (30.0)
Self-rated academic performance	1 = Low	1,094 (25.0)
	2 = Middle	2,212 (50.6)
	3 = High	1,066 (24.4)
PS	M ± SD	19.568 ± 8.604
PSQI	M ± SD	6.788 ± 2.488
SI	M ± SD	14.717 ± 5.587
PE	M ± SD	23.012 ± 8.716

### 2.2 Measurement instruments

#### 2.2.1 Perceived stress

Perceived stress (PS) was assessed using the Simplified Chinese version of the 10-item Perceived Stress Scale (PSS-10) ((37, 38)). Evidence for the factorial validity of the PSS-10 among Chinese adolescents has also been reported (39). The scale assesses the extent to which respondents appraised their lives as unpredictable, uncontrollable, and overloaded during the preceding month. It comprises six negatively worded items and four positively worded items, with the latter reverse-scored. Items are rated on a five-point Likert scale ranging from 0 (never) to 4 (always), yielding a total score of 0–40; higher scores indicate greater perceived stress. In this study, the PSS-10 demonstrated good internal consistency (Cronbach’s *α* = 0.834) and sampling adequacy (KMO = 0.921). The CFA results yielded mixed evidence regarding model fit. The χ^2^/df ratio was 8.238, exceeding conventional recommended values, whereas the RMSEA (0.041), SRMR (0.027), CFI (0.975), and TLI (0.968) were within commonly recommended ranges. Given the large sample size (*N* = 4,372) and the sensitivity of χ^2^-based statistics to sample size and minor model misspecification, model fit was evaluated across multiple indices rather than on the χ^2^/df ratio alone. Taken together, the results provided generally acceptable support for the measurement model, although the elevated χ^2^/df ratio indicates some degree of model–data misfit and warrants cautious interpretation. The AVE and CR values were 0.535 and 0.834, respectively.

#### 2.2.2 Sleep quality

Sleep quality (PSQI) was measured using the Pittsburgh Sleep Quality Index developed by Buysse (17), which evaluates various aspects of sleep, including subjective sleep quality, sleep onset latency, sleep duration, sleep efficiency, sleep disturbances, use of sleep medication, and daytime dysfunction. The scale comprises 7 components, with a total score range of 0–21; higher scores indicate poorer sleep quality. In this study, the PSQI demonstrated good internal consistency (Cronbach’s *α* = 0.813) and sampling adequacy (KMO = 0.890). CFA results indicated satisfactory fit: χ^2^/df = 3.786, RMSEA = 0.032, SRMR = 0.027, CFI = 0.979, TLI = 0.962.

#### 2.2.3 Suicidal ideation

Suicidal ideation was assessed using the Negative Suicidal Ideation subscale of the Positive and Negative Suicide Ideation Inventory (PANSI) developed by Osman (40). This subscale contains 7 items, evaluating the frequency and severity of suicidal thoughts, death-related thoughts, and associated negative cognitions over a specific period. Items are rated on a 5-point Likert scale (1 = never, 5 = always), with total scores ranging from 7 to 35; higher scores indicate higher levels of SI. In this study, the subscale demonstrated good internal consistency (Cronbach’s *α* = 0.802) and sampling adequacy (KMO = 0.879). CFA indicated acceptable fit: χ^2^/df = 2.646, RMSEA = 0.040, SRMR = 0.036, CFI = 0.935, TLI = 0.963, with AVE = 0.568 and CR = 0.803.

#### 2.2.4 Physical exercise

Physical exercise (PE) was assessed using the Physical Activity Rating Scale-3 (PARS-3) revised by Liang (41). The PARS-3 is an established behavioral exercise-volume index comprising three conceptually distinct components: exercise intensity, duration, and frequency. Exercise volume was calculated as Exercise Intensity × (Exercise Duration − 1) × Exercise Frequency, yielding a score ranging from 0 to 100, with higher scores indicating greater exercise volume. This standardized scoring procedure has been used in Chinese adolescent populations, with previous research providing evidence of temporal stability (42). Unlike items in a reflective psychometric scale, the three PARS-3 components jointly define exercise volume and are combined algorithmically rather than treated as interchangeable manifestations of a single latent construct. Accordingly, CFA and reflective-measurement indices, including AVE and CR, were not applicable and were therefore not estimated.

### 2.3 Covariates

In this study, gender, grade level, only-child status, boarding status, and self-reported academic performance were included as covariates to control for potential confounding effects of demographic factors on the relationships among the main variables. These covariates were selected *a priori* because gender and grade level may be related to differences in academic stress and sleep-related experiences (43, 44); only-child status and boarding status may reflect differences in family structure, living arrangements, and access to daily support; and self-reported academic performance may capture variation in perceived academic demands and academic self-evaluation. In the PROCESS path models, gender, grade, only-child status, boarding status, and self-rated academic performance were controlled as covariates. In the supplementary machine-learning analyses, age, family economic status, parental marital status, and PE category were additionally included to improve the informational completeness of the prediction models. The inclusion of gender and boarding status as covariates was intended to reduce potential confounding within the achieved sample; it does not correct the demographic imbalance introduced by non-probability school recruitment or render the estimates population-representative.

### 2.4 Data processing and analysis

Data processing and statistical analyses were conducted using SPSS 26.0, R 4.3.1, and Python. Normality tests and descriptive statistics were performed for continuous variables, whereas frequencies and percentages were calculated for categorical variables. Common method bias was assessed using Harman’s single-factor test, followed by reliability and validity assessments of the primary measurement instruments. Gender, grade level, only-child status, boarding status, and self-reported academic performance were included as covariates in the subsequent models.

Pearson correlations were first calculated to examine the overall associations among PS, PSQI, PE, and SI. Mediation was tested using PROCESS Model 4, and the hypothesized moderation of the PS–SI and PSQI–SI paths by PE was tested using Model 15. To examine the alternative possibility of first-stage moderation, a supplementary expanded conditional process model additionally included the PS × PE interaction in the equation predicting PSQI while retaining the PS × PE and PSQI × PE interactions in the equation predicting SI. Because moderation of the PS–PSQI path was not specified in H3 or H4, this analysis was treated as supplementary rather than confirmatory. All continuous focal variables were Z-standardized before the PROCESS analyses. Indirect and conditional indirect effects were estimated using 5,000 bootstrap resamples, with statistical significance determined by 95% CIs that excluded zero.

As a sensitivity analysis, poor sleeper status was defined separately using PSQI scores greater than 5 and greater than 7 (17, 45). Because the resulting mediator was binary, logistic regression was used to model poor sleeper status, whereas linear regression was used to model SI as a function of PS and poor sleeper status. PS and SI were standardized, the covariate specification was identical to that of the primary mediation model, and indirect associations corresponding to a one-standard-deviation increase in PS were estimated using marginal standardization with 5,000 nonparametric bootstrap resamples.

Supplementary machine-learning analyses were conducted in Python to assess the predictive contributions of the study variables to total SI scores (SI_T). Predictors included demographic variables, PS_T, PSQI_T, PE_T, PE category, and the PS × PE and PSQI × PE interaction terms; SI1–SI7 were excluded to prevent outcome leakage. The sample was randomly divided into training and hold-out test sets at an 80:20 ratio, with five-fold cross-validation conducted within the training set. Ridge regression and gradient boosting regression were evaluated using RMSE, MAE, and R^2^. Predictor contributions were estimated using a five-fold test-only Monte Carlo SHAP procedure and expressed as each predictor’s mean absolute SHAP value relative to the sum across all predictors.

Network analysis complemented the total-score-based path and prediction models by treating scale items and dimensions as nodes, thereby identifying within- and cross-construct conditional associations, central nodes, and bridge nodes (46, 47). A Gaussian graphical model with graphical LASSO regularization was estimated using the qgraph, bootnet, and networktools packages in R (48). The regularization parameter was selected using the extended Bayesian information criterion (*γ* = 0.50), and the network was visualized using the Fruchterman–Reingold algorithm. Node centrality was evaluated using strength and expected influence, whereas bridge centrality was assessed using bridge strength and bridge expected influence. Nonparametric bootstrapping was used to estimate 95% CIs for edge weights, and case-dropping bootstrapping was used to assess centrality stability. CS coefficients greater than 0.25 and 0.50 were considered acceptable and good, respectively. All statistical tests were two-tailed, with *p* < 0.05 indicating statistical significance.

## 3 Results

### 3.1 Common method bias test

After data collection, common method bias was assessed using Harman’s single-factor test. All items from the main variables were included in an unrotated exploratory factor analysis. Results indicated that the first factor accounted for 27.30% of the total variance, which is below the 40% threshold, suggesting that common method bias was not a serious concern in this study.

### 3.2 Correlation analysis

As shown in Table 2, PS was significantly positively correlated with PSQI and SI (*r* = 0.503 and 0.444, both *p* < 0.001), and PSQI was significantly positively correlated with SI (*r* = 0.575, *p* < 0.001). PE was significantly negatively correlated with PS, PSQI, and SI (*r* = −0.138, −0.306, −0.286, all *p* < 0.001). These results indicate that higher levels of PS and poorer PSQI are associated with elevated SI, whereas higher levels of PE may serve a protective role, providing a rationale for subsequent mediation and moderated mediation analyses.

**Table 2:** Correlations among the main study variables.

Variable	PS	PSQI	SI	PE
PS	1			
PSQI	0.503***	1		
SI	0.444***	0.575***	1	
PE	−0.138***	−0.306***	−0.286***	1

***p < 0.001.

### 3.3 Mediation model testing of PSQI

After controlling for gender, grade, only-child status, boarding status, and self-reported academic performance, PS was significantly positively associated with PSQI (*β* = 0.486, *p* < 0.001), with the model explaining 26.7% of the variance. When both PS and PSQI were included in the model, PS (*β* = 0.201, *p* < 0.001) and PSQI (*β* = 0.460, *p* < 0.001) were both significantly positively associated with SI, with the model explaining 37.0% of the variance. In a model excluding PSQI, the total effect of PS on SI was also significant (*β* = 0.425, *p* < 0.001), with 21.5% of the variance explained. Compared with the total effect model, inclusion of PSQI reduced the direct effect of PS on SI from 0.425 to 0.201, while remaining statistically significant, indicating that PSQI partially mediates the relationship between PS and SI. Overall, higher PS is associated with higher SI, and this association may also operate indirectly through poorer PSQI. Detailed results are presented in Table 3.

**Table 3:** Regression analysis of the mediating role of PSQI.

Outcome variables	Predictive variables	*R*	*R^2^*	*F*	*β*	95% CI
PSQI	PS	0.516	0.267	264.590***	0.486***	[0.460, 0.512]
	Gender				0.147***	[0.094, 0.199]
	Grade				0.160***	[0.109, 0.211]
	Only child				−0.044	[−0.095, 0.008]
	Boarding				0.026	[−0.029, 0.082]
	Academic				−0.074***	[−0.111, −0.038]
SI	PS	0.608	0.370	366.400***	0.201***	[0.174, 0.228]
	PSQI				0.460***	[0.433, 0.488]
	Gender				0.152***	[0.104, 0.201]
	Grade				0.012	[−0.035, 0.060]
	Only child				−0.020	[−0.068, 0.028]
	Boarding				0.011	[−0.040, 0.062]
	Academic				−0.066***	[−0.100, −0.032]
SI	PS	0.464	0.215	199.186***	0.425***	[0.398, 0.451]
	Gender				0.220***	[0.166, 0.274]
	Grade				0.086**	[0.033, 0.139]
	Only child				−0.040	[−0.094, 0.013]
	Boarding				0.023	[−0.034, 0.081]
	Academic				−0.100***	[−0.138, −0.062]

*p < 0.05, ** *p* < 0.01, *** *p* < 0.001.

Bootstrap analyses indicated that the total effect of PS on SI was significant (*β* = 0.425, Boot 95% CI [0.397, 0.452]). After including PSQI, the direct effect remained significant (*β* = 0.201, Boot 95% CI [0.174, 0.228]), and the indirect effect via PSQI was also significant (*β* = 0.224, Boot 95% CI [0.205, 0.243]). The indirect effect accounted for 52.65% of the total effect, suggesting that PSQI may serve as an important statistical pathway linking PS to SI. Detailed results are presented in Table 4.

**Table 4:** Bootstrap analysis of mediating effects of PSQI.

Effect	Effect value	Boot SE	Boot 95% CI	Proportion of effects (%)
Total effect: ZPS → ZSI	0.425***	0.014	[0.397, 0.452]	100.00
Direct effect: ZPS → ZSI	0.201***	0.014	[0.174, 0.228]	47.35
Indirect effect: ZPS → ZPSQI → ZSI	0.224***	0.010	[0.205, 0.243]	52.65

***p < 0.001.

Sensitivity analyses were further conducted using two threshold-based definitions of poor sleeper status. Based on PSQI scores greater than 5 and greater than 7, 2,422 (55.40%) and 1,720 (39.34%) participants, respectively, were classified as poor sleepers. For the >5 cutoff, a one-standard-deviation increase in PS was associated with higher odds of poor sleeper status (OR = 2.531, 95% CI [2.349, 2.727]), and poor sleeper status was associated with higher SI after adjustment for PS and the covariates (*β* = 0.607, 95% CI [0.552, 0.662]). The corresponding indirect association was 0.121 (Boot 95% CI [0.109, 0.134]). Using the more stringent >7 cutoff produced a similar pattern: PS was associated with higher odds of poor sleeper status (OR = 2.583, 95% CI [2.393, 2.787]), which was in turn associated with higher SI (*β* = 0.691, 95% CI [0.635, 0.746]). The corresponding indirect association was 0.159 (Boot 95% CI [0.141, 0.178]). These findings were consistent with the primary analysis using continuous PSQI scores, indicating that the observed indirect association was robust across alternative operationalizations of sleep quality.

### 3.4 Moderated effects of PE

Results from Model 15 indicated that PE moderated the relationships among PS, PSQI, and SI. PS was significantly positively associated with PSQI (*β* = 0.486, *p* < 0.001). In the SI model, both PS (*β* = 0.209, *p* < 0.001) and PSQI (*β* = 0.378, *p* < 0.001) were significantly positively associated with SI, while PE was significantly negatively associated with SI (*β* = −0.197, *p* < 0.001). Interaction effects revealed that PS × PE (*β* = −0.078, *p* < 0.001) and PSQI × PE (*β* = −0.144, *p* < 0.001) were both significant, indicating that at higher levels of PE, the positive associations between PS and SI and between PSQI and SI were attenuated. The moderating effect of PE on the PSQI–SI association was relatively stronger. Detailed results are presented in Table 5.

**Table 5:** Regression analysis of moderated mediation effects.

Outcome variables	Predictive variables	*R*	*R^2^*	*F*	*β*	95% CI
PSQI	PS	0.516	0.267	264.590***	0.486***	[0.460, 0.512]
	Gender				0.147***	[0.094, 0.199]
	Grade				0.160***	[0.109, 0.211]
	Only child				−0.044	[−0.095, 0.008]
	Boarding				0.027	[−0.029, 0.082]
	Academic				−0.075***	[−0.111, −0.038]
SI	PS	0.642	0.411	304.924***	0.209***	[0.182, 0.235]
	PSQI				0.378***	[0.350, 0.406]
	PE				−0.197***	[−0.223, −0.170]
	PS × PE				−0.078***	[−0.103, −0.052]
	PSQI × PE				−0.144***	[−0.174, −0.114]
	Gender				0.119***	[0.071, 0.166]
	Grade				0.016	[−0.030, 0.062]
	Only child				−0.014	[−0.060, 0.032]
	Boarding				0.005	[−0.045, 0.055]
	Academic				−0.071***	[−0.104, −0.038]

***p < 0.001.

To further clarify the moderating role of PE, results are presented in Table 6 and Figures 2–4. Conditional direct effect analyses indicated that the association between PS and SI was significant at low, medium, and high levels of PE, but the effect size gradually decreased as PE increased, with values of 0.276, 0.241, and 0.136, respectively. Similarly, the association between PSQI and SI also declined with higher PE levels, with conditional effects of 0.502, 0.437, and 0.244 at low, medium, and high PE, respectively. Conditional indirect effect analyses showed that the indirect effect of PS on SI through PSQI was significant across all PE levels and exhibited a decreasing trend. The indirect effects were 0.244, 0.212, and 0.118 at low, medium, and high PE, respectively, with all Bootstrap 95% confidence intervals not including zero. The moderated mediation index was −0.070, Bootstrap 95% CI [−0.086, −0.054], indicating that PE significantly moderated the indirect pathway from PS through PSQI to SI. Overall, higher levels of PE attenuate the risk associations among PS, poor PSQI, and SI.

**Table 6:** Conditional indirect effects by PE.

Moderator level	Conditional direct effect PS → SI	Conditional effect PSQI → SI	Indirect effect PS → PSQI → SI	Boot 95% CI
Low PE	0.276***	0.502***	0.244	[0.221, 0.267]
Medium PE	0.241***	0.437***	0.212	[0.193, 0.232]
High PE	0.136***	0.244***	0.118	[0.097, 0.141]
Index of moderated mediation			−0.070	[−0.086, −0.054]

Low PE, Medium PE, and High PE correspond to the 16th, 50th, and 84th percentiles of the standardized PE distribution in the sample, respectively. ***p < 0.001.

**Figure 2:**
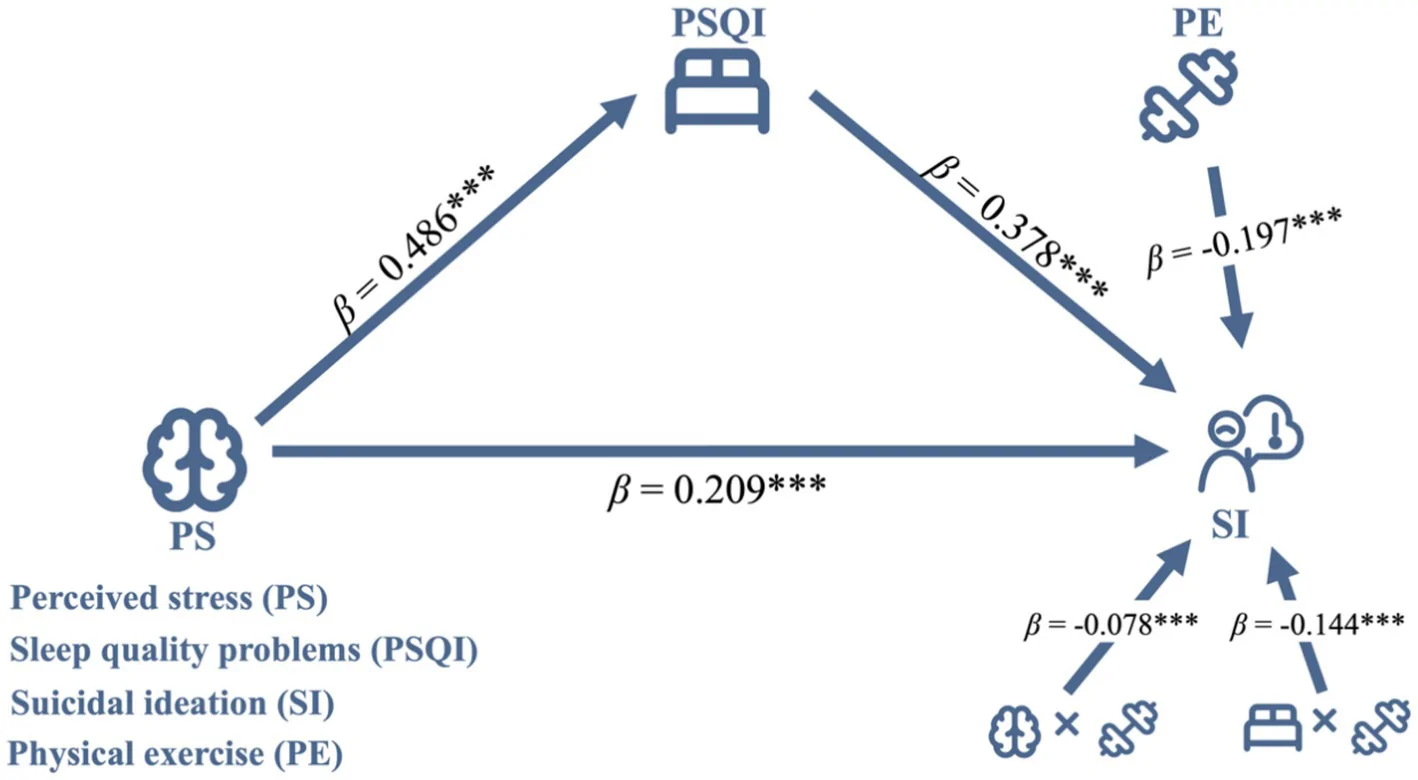
Moderated mediation model.

**Figure 3:**
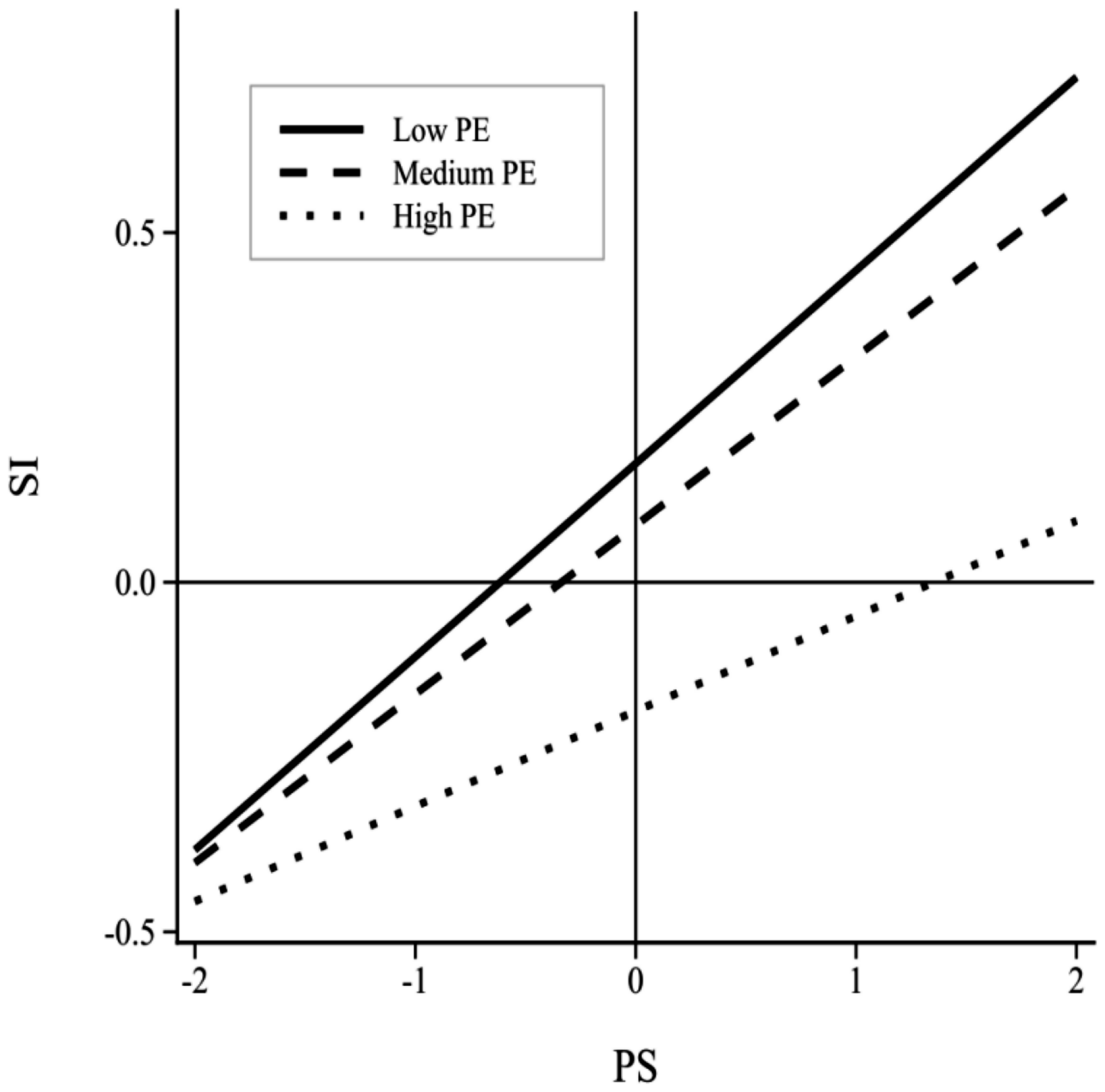
Moderating effect of PE on the relationship between PS and SI.

**Figure 4:**
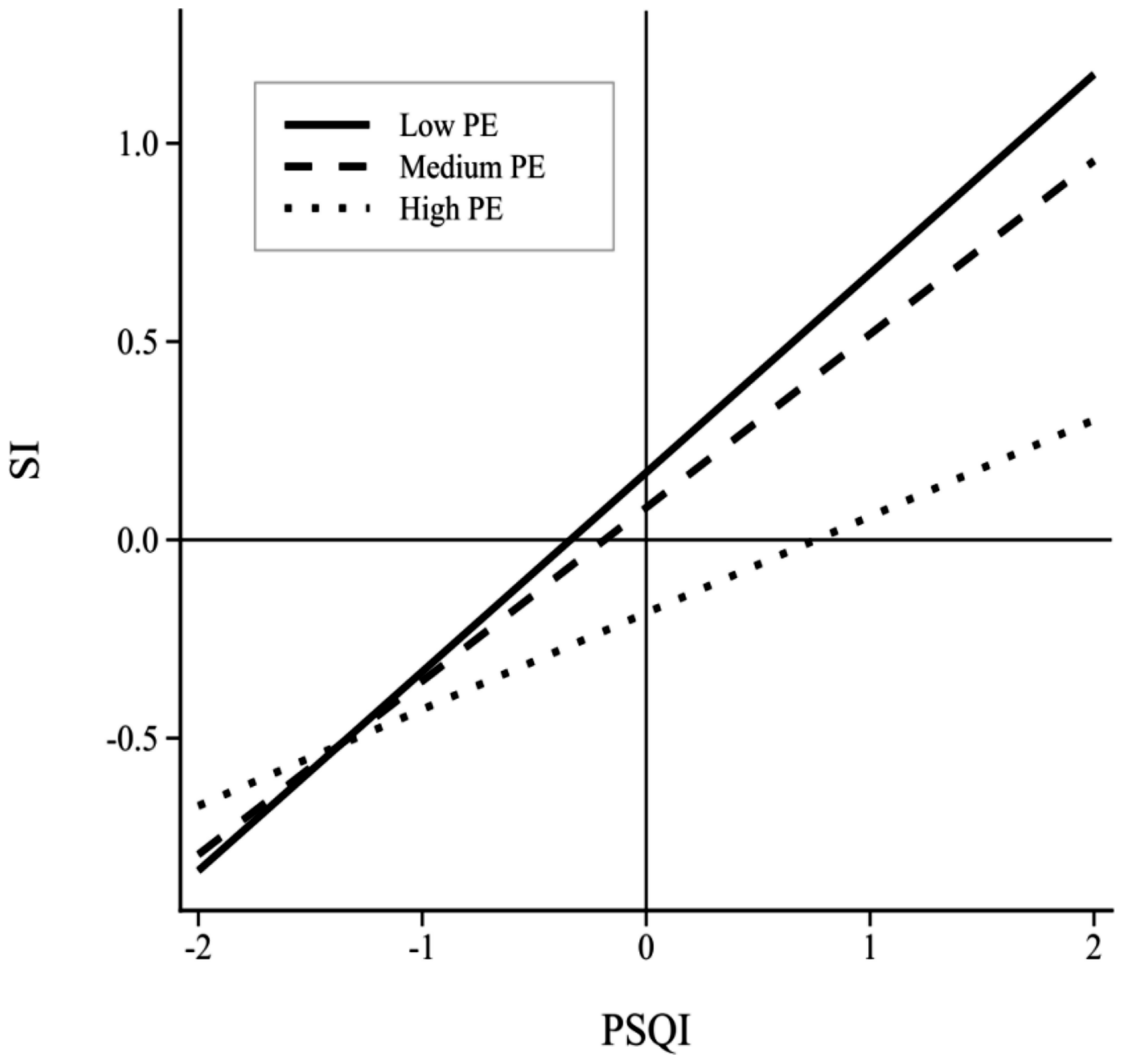
Moderating effect of PE on the relationship between PSQI and SI.

As a supplementary analysis, we extended Model 15 by including the PS × PE interaction in the equation predicting PSQI. This first-stage interaction was not statistically significant (*β* = −0.018, *SE* = 0.013, *t* = −1.385, *p* = 0.166, 95% CI [−0.043, 0.007]), providing no evidence that the association between PS and PSQI varied across levels of PE. Within the expanded model, the PS × PE interaction predicting SI (*β* = −0.078, *SE* = 0.013, *t* = −5.889, *p* < 0.001, 95% CI [−0.103, −0.052]) and the PSQI × PE interaction predicting SI (*β* = −0.144, *SE* = 0.015, *t* = −9.414, *p* < 0.001, 95% CI [−0.174, −0.114]) remained significant. Thus, including the first-stage interaction did not alter the inferential conclusions concerning the two hypothesized interaction effects on SI, whereas the supplementary analysis did not support moderation of the PS–PSQI pathway.

### 3.5 Machine-learning prediction and SHAP interpretation

To further examine the predictive value of the main variables for suicidal ideation (SI) from a prediction-oriented perspective, the total SI score (SI_T) was specified as a continuous outcome variable. The predictors included sex, grade, age, only-child status, boarding status, family economic status, parental marital status, self-rated academic performance, PE category, total perceived stress score (PS_T), total sleep quality score (PSQI_T), total physical exercise score (PE_T), and the PS × PE and PSQI × PE interaction terms. To avoid outcome leakage, the individual SI items (SI1–SI7) were excluded from the predictor set. The sample was randomly divided into a training set and a hold-out test set at an 80:20 ratio, and five-fold cross-validation was performed within the training set. Ridge regression and gradient boosting tree regression were used as predictive models. Variable contributions were estimated using a five-fold test-only Monte Carlo SHAP procedure. This analysis was intended to provide complementary evidence regarding the predictive contribution of the study variables and was not used to infer causal relationships (Table 7).

**Table 7:** Machine-learning model performance for predicting suicidal ideation.

Model	Training n	Test *n*	5-fold CV RMSE	5-fold CV MAE	5-fold CV R^2^	Holdout RMSE	Holdout MAE	Holdout R^2^
Gradient boosting	3,497	875	4.330 ± 0.114	3.430 ± 0.106	0.400 ± 0.026	4.254	3.357	0.412
Ridge regression	3,497	875	4.282 ± 0.157	3.393 ± 0.136	0.413 ± 0.037	4.249	3.346	0.413

The model performance results indicated that both algorithms showed acceptable predictive ability. In the hold-out test set, the gradient boosting tree model achieved an R^2^ of 0.412, an RMSE of 4.254, and an MAE of 3.357. The ridge regression model showed highly comparable performance, with an R^2^ of 0.413, an RMSE of 4.249, and an MAE of 3.346. These results suggest that the current set of predictors explained approximately 41% of the variance in total suicidal ideation (SI) scores in the hold-out test set.

SHAP contribution rates were further calculated based on the gradient boosting tree model. The results showed that PSQI were the most influential predictor, accounting for 33.70% of the total SHAP contribution. This was followed by perceived stress (PS; 15.49%), the PSQI × PE interaction term (9.86%), PE (9.58%), and the PS × PE interaction term (7.16%). The SHAP beeswarm plot further indicated that higher levels of PSQI-related sleep problems and PS generally contributed to higher predicted SI scores, whereas higher levels of PE were overall associated with lower predicted SI scores. These findings provide prediction-oriented evidence supporting the key roles of PS, PSQI, and PE in the formation mechanism of SI identified in the main analyses (Figure 5).

**Figure 5:**
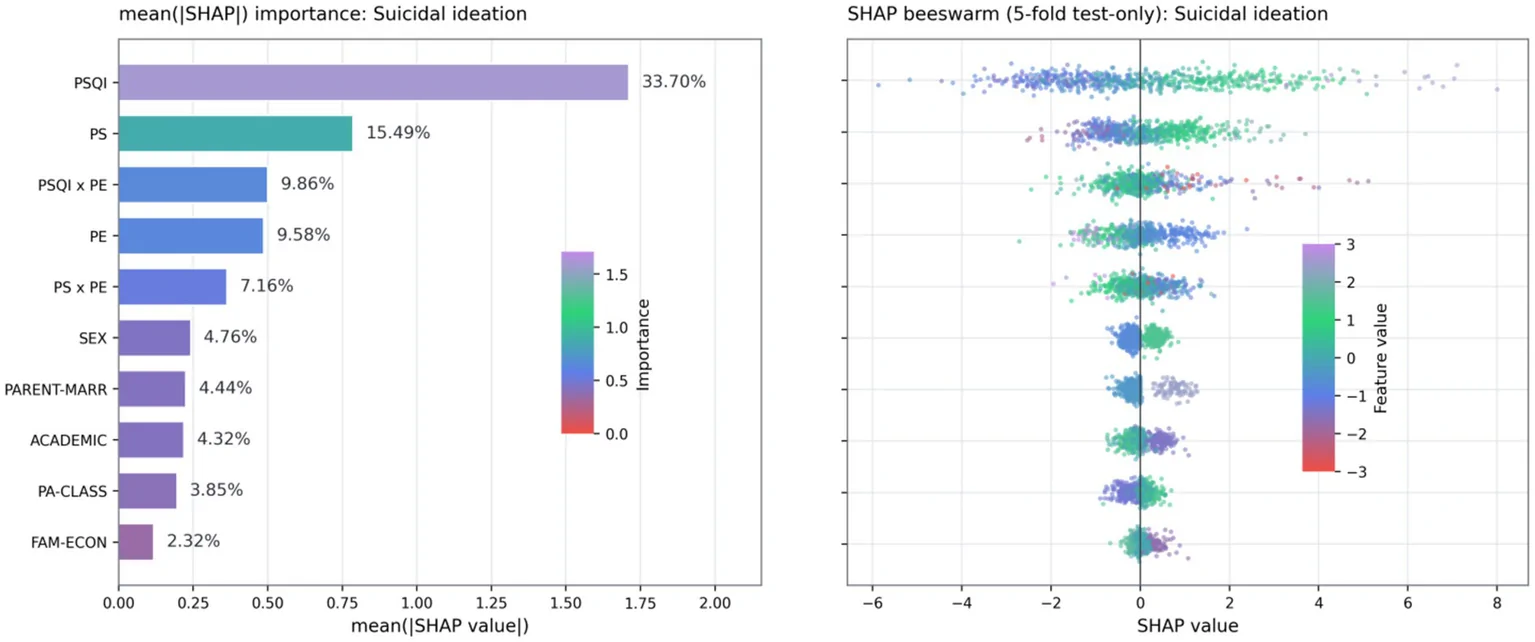
Feature importance and SHAP explanations for the gradient boosting model predicting suicidal ideation.

### 3.6 Network analysis of variables

#### 3.6.1 Primary network structure

The primary network results (Figure 6a) indicated that PS, PSQI, SI, and PE each formed relatively distinct internal clusters. Within the PS nodes, connections were pronounced, suggesting high co-occurrence among components of stress experiences such as uncontrollability, tension, coping difficulties, and cumulative stress. The SI nodes also exhibited strong internal connectivity, indicating a high co-occurrence among different forms of suicide-related thoughts and negative cognitions. PSQI dimensions were tightly connected, supporting the multidimensional co-occurrence characteristics of sleep quality. Similarly, the three PE nodes showed strong interconnections, reflecting consistency among PE intensity, duration, and frequency. The bridge network results (Figure 6b) revealed that cross-cluster connections were primarily concentrated among PSQI, SI, PS, and PE. Notably, PSQI nodes displayed multiple cross-cluster connections with SI nodes, suggesting that PSQI may serve as a key network link connecting PS and SI. PE nodes also exhibited bridging connections with PSQI, SI, and some PS nodes, indicating that PE may participate in the regulatory structure of the PS–PSQI–SI network. Overall, in final-year secondary school students, PS, PSQI, SI, and PE form an interconnected psychological-behavioral network through multiple conditional associations at the node level (see Figures 6a,b).

**Figure 6:**
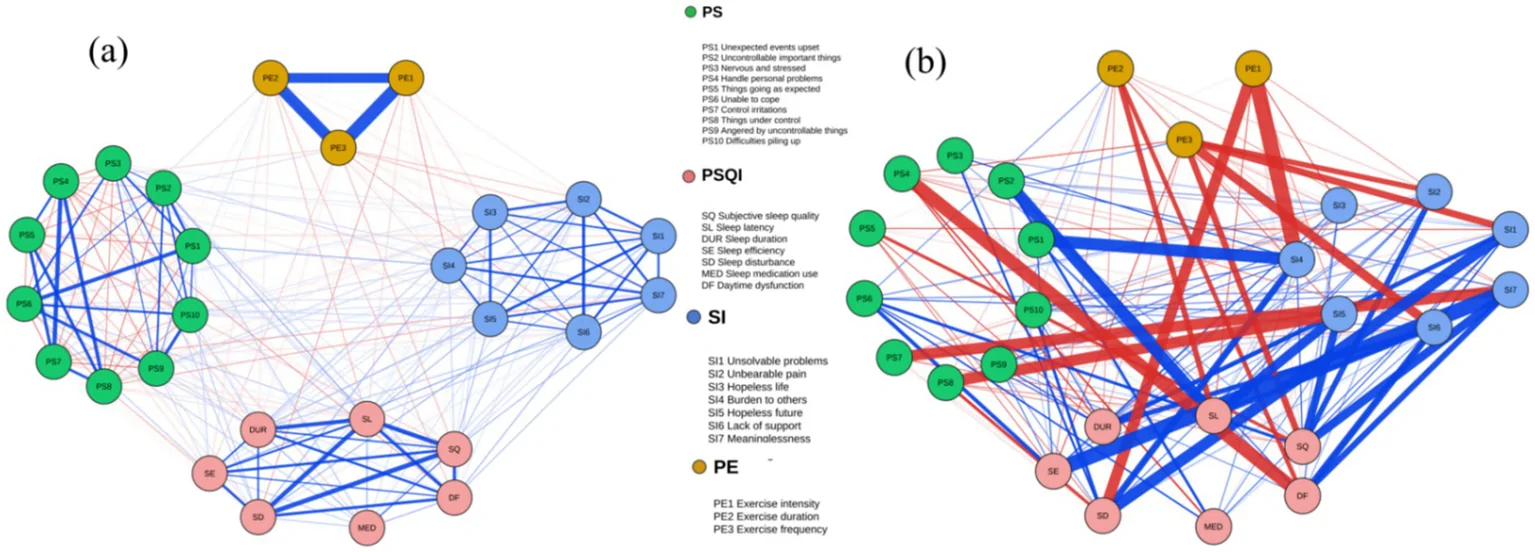
Node-level network structure. Panel **(a)** shows the full estimated node-level network, whereas panel **(b)** shows the bridge-only network retaining only cross-community edges. Blue edges indicate positive associations among nodes, whereas red edges indicate negative partial correlations. Node labels are defined as follows. PSS-10 nodes: PS1, distress caused by unexpected events; PS2, inability to control important things; PS3, feelings of nervousness and stress; PS4, confidence in handling personal problems; PS5, perception that things are going as intended; PS6, inability to cope with required tasks; PS7, ability to control irritations; PS8, feeling that things are under control; PS9, anger about events outside one’s control; and PS10, accumulation of difficulties that feel difficult to overcome. PSQI nodes: SQ, subjective sleep quality; SL, sleep latency; DUR, sleep duration; SE, habitual sleep efficiency; SD, sleep disturbances; MED, use of sleep medication; and DF, daytime dysfunction. PANSI negative-ideation nodes: SI1, suicidal thoughts related to unsolvable problems; SI2, suicidal thoughts related to unbearable pain; SI3, suicidal thoughts related to hopelessness about life; SI4, suicidal thoughts related to perceived burdensomeness; SI5, suicidal thoughts related to hopelessness about the future; SI6, suicidal thoughts related to a perceived lack of understanding or support; and SI7, suicidal thoughts related to the perceived meaninglessness of life. PARS-3 nodes: PE1, exercise intensity; PE2, exercise duration; and PE3, exercise frequency.

#### 3.6.2 Core nodes and centrality results

Further centrality analyses indicated that SQ exhibited the highest strength (strength = 1.147, *z* = 1.859), followed by SL (strength = 1.103, *z* = 1.509), SD (strength = 1.056, *z* = 1.135), PS6 (strength = 1.031, *z* = 0.937), and PE3 (strength = 0.989, *z* = 0.607). These results identify SQ as the most strongly connected node in the overall network, with SL, SD, PS6, and PE3 also exhibiting relatively high connectivity. Consistent with the node-strength results, bridge-strength analysis likewise ranked SQ first (bridge strength = 0.321, *z* = 1.751), followed by SI4, SD, DUR, SL, and DF, indicating that sleep-related nodes and the perceived-burdensomeness component were prominent in cross-community connectivity. The convergence of node strength and bridge strength indicates that SQ was not only broadly connected in the network as a whole but also occupied the most prominent cross-community position, reinforcing its structural prominence across these complementary centrality dimensions. Bridge expected influence analysis indicated that PS6, PS2, PS9, and SI4 were on the positive end, whereas PE3 (−0.198, *z* = −2.010), PE1 (−0.173, *z* = −1.799), and PE2 (−0.123, *z* = −1.392) were on the negative end, suggesting that PE-related nodes may be inversely connected to the risk network. Overall, SQ, SL, SD, PS6, SI4, and PE-related nodes appear to represent key bridging points within the psychological–behavioral network of graduating-grade secondary school students. The bridge-centrality results are presented in Figure 7.

**Figure 7:**
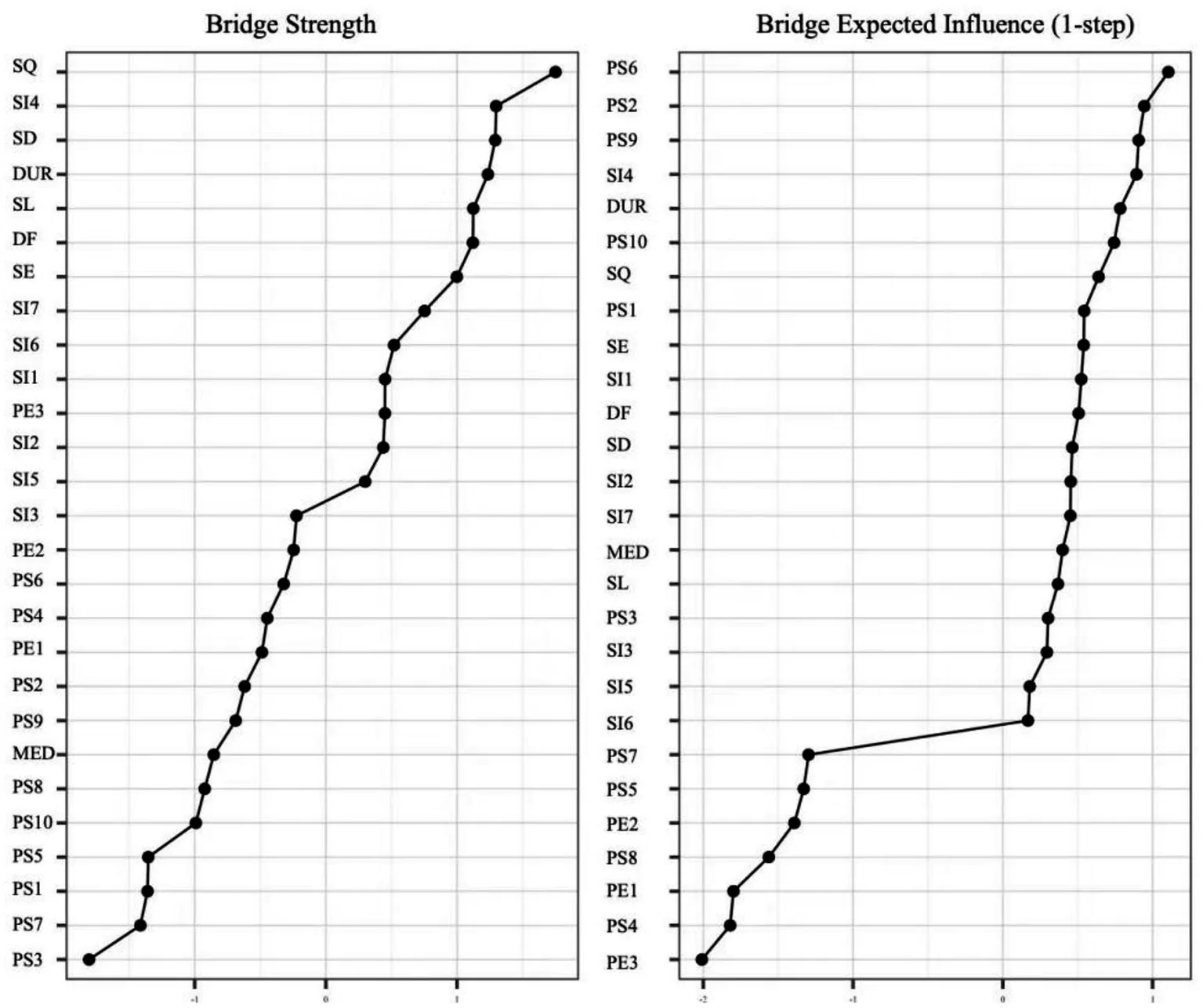
Bridge centrality indices of nodes in the PS–PSQI–SI–PE network.

#### 3.6.3 Network accuracy and stability estimation

Network stability analyses (Figure 8) indicated that the CS-coefficients for strength and expected influence were both 0.75, exceeding the recommended threshold of 0.50, suggesting that the centrality results are highly stable. Nonparametric bootstrap analyses showed relatively narrow 95% confidence intervals for the main edge weights, and the trends of the sample estimates were largely consistent with the bootstrap means, indicating that the estimates of key connections within the network are stable. Overall, the network structure in this study demonstrates acceptable edge-weight accuracy and good centrality stability, supporting the identification of key nodes based on centrality and bridge centrality metrics.

**Figure 8:**
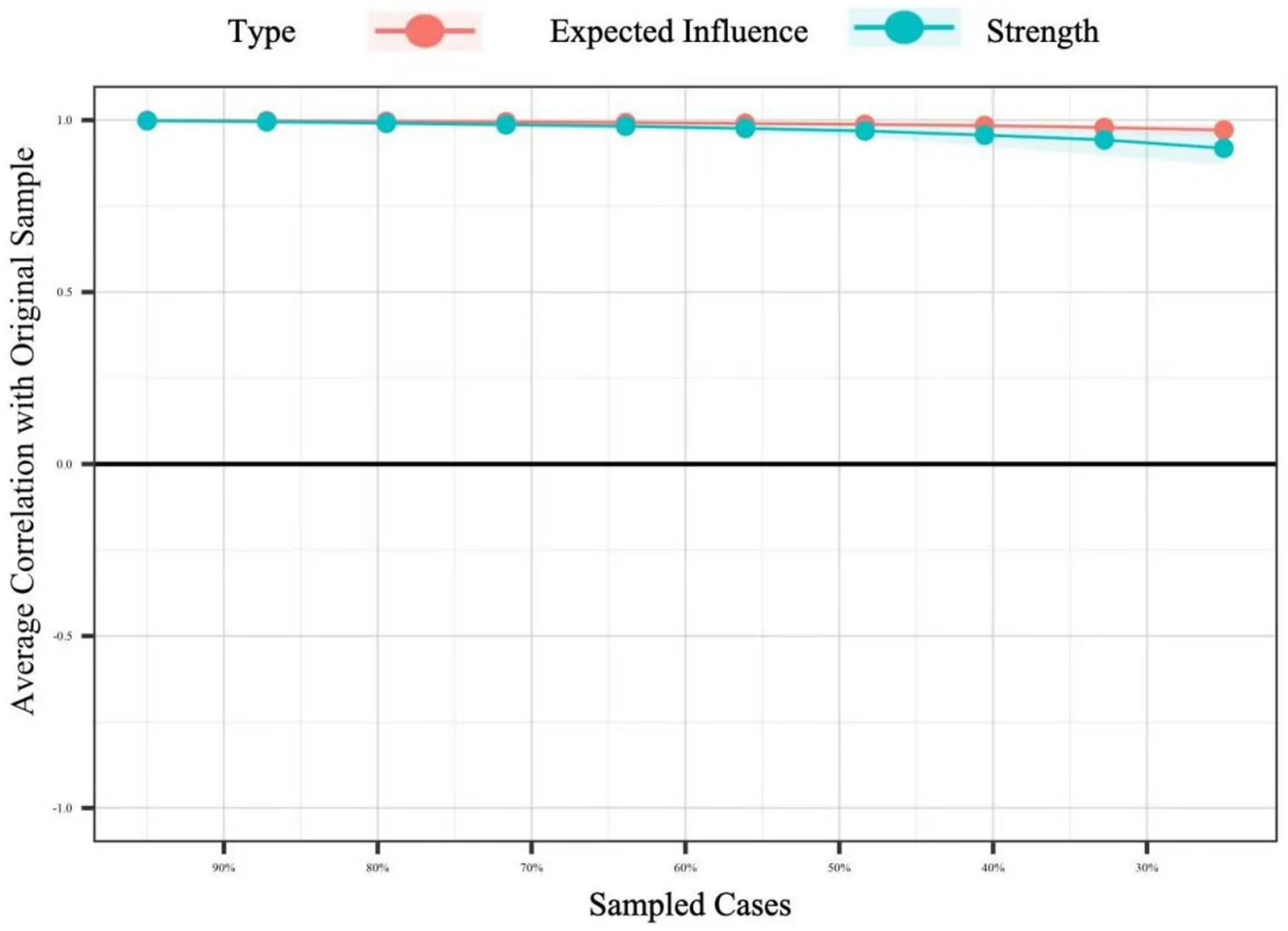
Case-dropping bootstrap analysis of strength centrality.

## 4 Discussion

This study examined the relationships among PS, PSQI, PE, and SI among Chinese graduating-grade secondary school students, and integrated evidence from path modeling, machine learning, and network analysis to provide complementary perspectives. The results showed that PS was significantly and positively associated with SI, and that PSQI partially mediated this association, suggesting that stressful experiences may be linked to SI partly through impaired sleep restoration. PE showed a potential protective role. Specifically, when PE levels were higher, the positive associations of PS and PSQI-related sleep problems with SI were relatively weaker, indicating that regular PE may help buffer the psychological risks associated with stress and poor sleep.

The supplementary machine-learning analyses provided further prediction-oriented support for these findings. Both ridge regression and gradient boosting tree models showed satisfactory performance in predicting total SI scores, explaining approximately 41% of the variance in the hold-out test set. SHAP results indicated that PSQI, PS, PE, and their interaction terms made substantial contributions to model prediction, with PSQI-related sleep problems showing the largest contribution. These findings suggest that SI is not explained by a single psychological factor alone, but is more likely associated with the combined effects of stress appraisal, insufficient sleep recovery, and limited health-behavior resources.

Network analysis further revealed that subjective sleep quality, sleep latency, sleep disturbances, stress-coping burden, perceived burdensomeness, and PE frequency were closely connected with the overall risk structure. These nodes may therefore represent key targets for psychological risk identification and intervention among graduating-grade secondary school students. Overall, this study not only confirmed the statistical associations among PS, PSQI, PE, and SI, but also demonstrated the importance of these variables in risk prediction and structural connectivity through machine-learning and network-analytic evidence. The findings provide empirical support for school-based mental health screening, sleep-support programs, stress-management strategies, and PE promotion.

### 4.1 Association between perceived stress and suicidal ideation

The positive association between PS and SI supported Hypothesis H1. Graduating-grade students are exposed to multiple stressors, including entrance examinations, academic ranking, family expectations, and uncertainty about future educational and career trajectories. These stressors are often persistent and closely tied to students’ self-evaluation (43, 49). When students perceive academic demands as exceeding their coping capacity, or when they feel that important events are difficult to control, they may be more likely to experience depleted psychological resources and report tension, helplessness, and a sense of failure (9). Thus, PS is not merely a subjective reflection of academic burden; it also captures students’ appraisal of personal control and the predictability of future outcomes. This finding is consistent with previous research on academic stress and adolescent SI. Prior studies have shown that academic and life stressors may be associated with SI through negative affect, low perceived control, or heightened PS, and that psychosocial risk factors can predict persistent SI among adolescents (7, 11, 14).

The network analysis further refined the association between PS and SI. The findings suggest that PS was not linked to SI as a broad and undifferentiated construct, but rather entered the risk network through several specific stress-related experiences. In particular, PS2, “unable to control important things”; PS6, “unable to cope”; PS9, “angered by uncontrollable events”; and PS10, “difficulties piling up,” appeared to reflect more than general academic pressure. These nodes capture students’ perceived loss of control, failed coping, and sustained psychological load. For graduating-grade secondary school students, such experiences may be more indicative of depleted psychological resources than the objective intensity of academic workload alone. Moreover, these stress-related nodes were positioned close to SI3, “life is hopeless”; SI5, “future is hopeless”; and SI4, “being a burden to others,” suggesting that stressful experiences may be connected with SI through negative future appraisal and impaired self-worth. In other words, when students persistently feel that events are uncontrollable, problems are unsolvable, or difficulties are accumulating, stress may no longer remain limited to tension or anxiety, but may gradually develop into hopelessness, perceived failure, and burdensomeness. These results extend the path-model findings by showing that the association between PS and SI is reflected not only in the positive relationship between total scores, but also in conditional connections among specific nodes.

Overall, PS is an important correlate of SI among graduating-grade secondary school students. In particular, perceived uncontrollability, inability to cope, and accumulated difficulties may more precisely indicate students’ psychological risk status. School-based mental health programs should therefore not only address the intensity of academic stress, but also attend to students’ subjective interpretation of stress, perceived control, and available coping resources.

### 4.2 The mediating role of sleep quality

PSQI partially mediated the association between PS and SI, supporting Hypothesis H2. Students with higher levels of PS were more likely to report sleep problems, and those with more severe sleep problems also showed higher levels of SI. After PSQI was included in the model, the direct effect of PS remained significant, indicating that PSQI explained part, but not all, of the association between PS and SI. Previous studies have shown that elevated stress is often accompanied by repetitive pre-sleep thinking, worry, and heightened physiological arousal, which are closely related to sleep latency and reduced sleep restoration (50, 51). This finding is also consistent with research on sleep and suicide risk (52). Prior evidence suggests that sleep disturbances are associated with adolescent SI, suicide plans, and suicide attempts, and that sleep problems may be linked to difficulties in emotion regulation and risk-related cognitive processing among adolescents (19, 34). The threshold-based sensitivity analyses yielded findings consistent with those obtained using continuous PSQI scores. Under both the >5 and >7 cutoffs, higher PS was associated with a greater likelihood of being classified as a poor sleeper, and poor sleeper status was associated with higher SI. These results strengthen the screening relevance of the findings while supporting the robustness of the primary continuous-score analysis. Nevertheless, PSQI-based classification reflects self-reported sleep disturbance and should not be interpreted as a clinical diagnosis.

The network analysis further clarified the association between PSQI and SI. The results suggest that PSQI was not connected with SI merely as a global total score, but entered the risk network through several specific sleep-related problems. In particular, subjective sleep quality, sleep latency, sleep disturbances, and daytime dysfunction appeared to reflect more than insufficient sleep duration. These nodes captured reduced sleep restoration, disrupted nocturnal rest, and impaired daytime functioning. For graduating-grade secondary school students, such sleep problems may indicate insufficient psychophysiological recovery resources, thereby limiting their capacity to buffer the psychological depletion caused by sustained academic stress. Moreover, these sleep-related nodes were closely connected with SI3, “life is hopeless”; SI5, “future is hopeless”; and SI4, “being a burden to others,” suggesting that PSQI-related problems may be associated with SI through weakened emotion regulation, accumulated fatigue, and intensified negative cognition. In other words, when students experience persistent difficulty falling asleep, sleep disturbances, or daytime dysfunction, poor sleep may no longer represent a purely physiological problem, but may further weaken their ability to cope with stress and intensify hopelessness, perceived failure, and burdensomeness. Thus, PSQI not only played a statistical mediating role between PS and SI, but also showed conditional connections with suicide-related cognitions at the node level.

Overall, these findings indicate that PSQI-related sleep problems constitute an important statistical pathway linking PS to SI. Poor subjective sleep quality, sleep latency, sleep disturbances, and daytime dysfunction may signal a state of heightened psychological and physiological depletion among students. Previous studies have also shown that adolescent sleep disturbances are closely associated with suicidal ideation and may represent important targets for early identification and intervention (34, 53). Therefore, school-based stress interventions should incorporate parallel assessment and support for sleep problems.

### 4.3 Moderating role of PE on the PS–SI relationship

PE moderated the association between PS and SI, supporting Hypothesis H3. Specifically, the positive association between PS and SI was weaker among students with higher levels of PE. This finding is consistent with the stress-buffering model, which suggests that regular PE may be associated with reduced negative cognitive responses under stressful conditions by improving emotional experience, enhancing self-efficacy, and providing a controllable daily activity context (54–56). This result also aligns with evidence supporting the protective role of PE in adolescent mental health. Previous studies have shown that PE is associated with lower risks of depression and SI, and that these effects may operate through self-esteem, social support, perceived physical competence, and emotion regulation (25, 57–59).

The network analysis further refined this finding. PE3, “PE frequency”; PE2, “PE duration”; and PE1, “PE intensity” appeared to have distinct meanings within the network, with PE frequency being particularly noteworthy. Frequency reflects whether students maintain a relatively stable exercise habit, duration indicates the degree of behavioral engagement, and intensity is more closely related to physiological arousal during exercise. Compared with occasional high-intensity activity, regular PE may be more conducive to establishing a stable rhythm of psychological and physiological recovery. It may also be associated with weaker risk connections involving PS6, “unable to cope”; PS10, “difficulties piling up”; SI3, “life is hopeless”; and SI5, “future is hopeless.” Therefore, PE may not simply reduce stress itself. Rather, it may buffer the association between PS and SI by providing regular activity, emotional release, and a sense of personal control.

Overall, these findings suggest that PE may serve as an important behavioral resource buffering the association between PS and SI. Higher levels of PE do not necessarily imply the absence of stress, but may help students maintain emotional recovery capacity and self-efficacy under high-pressure conditions. This interpretation is consistent with evidence that physical activity may reduce psychological distress through neurobiological regulation, enhanced resilience, and improved emotion regulation (25, 55). Therefore, preparation for entrance examinations among graduating-grade students should not excessively reduce the time allocated to necessary PE.

### 4.4 Moderating role of PE on the relationship between PSQI and SI

PE also moderated the association between PSQI and SI, supporting Hypothesis H4. Poorer PSQI was associated with higher levels of SI, but this association was weaker among students with higher levels of PE. Previous studies have suggested that PE may help improve emotional states, stabilize sleep–wake rhythms, and enhance psychological recovery capacity (26, 29, 60, 61). The present findings indicate that exercise participation may attenuate the strength of the association between sleep problems and SI.

This result further extends the sleep-mediated model. If sleep problems represent an important pathway linking PS to SI, then PE may be associated with weaker risk transmission by enhancing recovery-related resources. For students with poor PSQI, school-based support should therefore not be limited to sleep-hygiene education, but may also incorporate regular, moderate, and sustainable PE programs. Such an approach is more consistent with the association structure observed in this study among stress, sleep, exercise, and SI. From a mechanistic perspective, PE may not directly eliminate sleep problems; rather, it may be related to students’ capacity to maintain emotional stability and cognitive control under conditions of poor sleep. Previous research has also suggested that exercise is involved in circadian rhythm regulation and has stable protective associations with depression and anxiety (26, 61). The supplementary analysis did not provide evidence that PE moderated the association between PS and PSQI, whereas the PS × PE and PSQI × PE interactions predicting SI remained significant in the expanded model. Accordingly, evidence for moderation by PE was observed for the two paths directly involving SI, but not for the first-stage PS–PSQI pathway. This pattern should not be interpreted as evidence that PE cannot influence stress-related sleep processes, as cross-sectional between-person data may not capture delayed or within-person moderation. Longitudinal studies using repeated assessments of PS, PE, and sleep are needed to examine this possibility more directly.

The network analysis further refined this finding. Subjective sleep quality, sleep latency, sleep disturbances, and daytime dysfunction reflected specific manifestations of insufficient sleep restoration and impaired daytime functioning. In contrast, PE frequency, PE duration, and PE intensity represented different levels of behavioral exercise resources. Among these, PE frequency may be particularly informative because it reflects whether students maintain a stable exercise habit. Compared with occasional high-intensity activity, regular PE may be more beneficial for maintaining physiological rhythms, relieving fatigue, and improving emotional states, thereby weakening the links between sleep problems and suicide-related cognitions such as SI3, “life is hopeless”; SI4, “being a burden to others”; and SI5, “future is hopeless.” Thus, the protective role of PE may be reflected not only in its potential contribution to better sleep, but also in the additional emotion-regulation and psychophysiological recovery resources it provides when students experience poor sleep. For graduating-grade secondary school students, prolonged reduction of PE time during examination preparation may therefore weaken their ability to cope with sleep insufficiency and psychological fatigue.

Overall, these findings suggest that PE has protective relevance in the association between PSQI-related sleep problems and SI. Regular PE may not directly resolve sleep problems, but it may be associated with better emotional stability, recovery rhythms, and daytime functioning, corresponding to a weaker sleep problem–SI association. Accordingly, mental health support for graduating-grade students should integrate sleep management with PE promotion.

### 4.5 Supplementary implications of the machine-learning prediction results

The machine-learning results complemented the preceding path-model findings from a prediction-oriented perspective. Both the ridge regression and gradient boosting tree models achieved an R^2^ of approximately 0.41 in the hold-out test set, indicating that PS, PSQI, PE, and their interaction terms had meaningful out-of-sample predictive value for SI. The SHAP results showed that PSQI-related sleep problems made the largest contribution, followed by PS, the PSQI × PE interaction term, PE, and the PS × PE interaction term. This ranking was generally consistent with the mediation and moderation findings, suggesting that PSQI is not only an important link between PS and SI, but also the most informative feature for identifying students with elevated SI in the prediction model.

The SHAP results further indicated that PSQI-related sleep problems had the highest predictive contribution, clearly exceeding that of the other variables. This finding is consistent with previous evidence indicating that sleep disturbance is a robust correlate of adolescent suicidal ideation and may provide an accessible indicator for school-based screening (34, 53). This suggests that PSQI may represent one of the most sensitive risk signals for identifying SI among graduating-grade secondary school students. This finding aligns closely with the mediation model. PSQI was not only a statistical mediator in the association between PS and SI, but also the most influential predictor in the machine-learning model. In other words, sleep problems may capture insufficient psychophysiological recovery, impaired emotion regulation, and depleted psychological resources under prolonged academic stress, making them a more proximal marker of SI risk than stress perception alone.

When interpreted together with the network analysis, the high-contribution variables identified by machine learning were not merely abstract total-score indicators, but could be further traced to specific risk-related nodes. Subjective sleep quality, sleep latency, sleep disturbances, inability to cope, accumulated difficulties, being a burden to others, and PE frequency may jointly constitute key indicators for SI risk identification among graduating-grade students. In this sense, the machine-learning results indicate which variables are more predictive of risk, whereas the network analysis further clarifies which specific components within these variables deserve closer attention. Their integration suggests that school-based mental health screening should not rely solely on SI scale scores, but should also consider multidimensional signals, including impaired sleep restoration, perceived loss of control under stress, burdensomeness, and insufficient regular PE. It should be noted that SHAP values reflect the contribution of variables to model prediction rather than causal effects. Therefore, the primary role of the machine-learning analysis in this study was to provide risk-identification and predictive evidence, which complements the mediation, moderation, and network analyses.

### 4.6 Limitations and future directions

Several limitations should be acknowledged. First, this study used a cross-sectional design, which precludes establishing the temporal ordering or causal direction of the associations among PS, PSQI, PE, and SI. Accordingly, the mediation, moderation, and moderated mediation findings should be interpreted as statistical associations rather than causal pathways. Relatedly, although the supplementary analysis did not identify a significant moderating effect of PE on the PS–PSQI association, this null interaction was derived from cross-sectional, between-person self-report data and should not be interpreted as definitive evidence that PE cannot modify stress-related changes in sleep. Future longitudinal within-person and intervention studies are needed to clarify the temporal relationships among these variables and determine whether changes in PE attenuate the association between stress and subsequent sleep problems.

Second, the sample was drawn from selected schools in Yunnan Province through school-level convenience recruitment. The achieved sample was notably unbalanced, with male students accounting for 61.9% of participants and boarding students accounting for 70.0%. These distributions may reflect the demographic and residential composition of the participating schools and classes and may limit the representativeness of the sample, particularly with respect to female and commuting students. Although gender and boarding status were included as covariates, statistical adjustment cannot restore population representativeness or eliminate selection bias arising from non-probability recruitment. Accordingly, the findings should not be interpreted as regional prevalence estimates, and the reported associations may be less transportable to student populations with different gender or boarding-status distributions. Future studies should employ stratified probability sampling or population-calibrated sampling weights and conduct prespecified subgroup or interaction analyses to determine whether the observed associations are consistent across gender and boarding-status groups.

Third, data were collected using self-report questionnaires administered collectively in classroom settings. Completing a highly sensitive measure of SI in the presence of classmates, even under anonymous conditions and standardized staff supervision, may have heightened participants’ concerns that their engagement with suicide-related content could be observed. This context may have increased social-desirability pressure and contributed to nondisclosure or underreporting of SI relative to more private, individually administered surveys. In addition, full anonymity prevented individual PANSI responses from being linked to specific students, thereby precluding score-triggered risk identification and follow-up. Consequently, students with elevated SI who neither displayed observable distress nor actively sought help may not have been identified. Future studies should adopt more private administration settings and ethics-approved, participant-initiated referral mechanisms that remain separate from anonymous research data, thereby better balancing privacy protection, response validity, and risk management.

Fourth, depressive symptoms were not assessed and therefore could not be included as a covariate. This is an important limitation because depression is strongly associated with SI and may also be associated with both perceived stress and sleep problems. Consequently, residual confounding by depressive symptoms cannot be excluded, and the estimated associations among PS, PSQI, and SI should not be interpreted as independent of depression. Other potentially important confounders—including anxiety, hopelessness, social support, school bullying, and family functioning—were also not assessed. Future studies should incorporate validated measures of depressive symptoms and other relevant psychosocial factors and examine whether the present associations remain robust after adjustment for these variables.

Fifth, the student data may have exhibited a nested structure at the school or class level, which was not explicitly modeled. In addition, cross-sectional network analysis identifies conditional associations among nodes but cannot establish their temporal or causal direction or demonstrate that intervening on a central node would necessarily produce changes in other nodes. Future research could apply multilevel models, longitudinal network analysis, and intervention-based network designs to examine the dynamic roles of key nodes in the development of SI.

Finally, although the machine-learning analyses provided complementary predictive evidence, the models were developed using cross-sectional self-report data and a specific sample partition. SHAP contribution rates represent model-based predictive contributions rather than causal effects. External validation in independent samples and the use of longitudinal prediction models are therefore required to evaluate the stability, transportability, and generalizability of these findings.

## 5 Conclusion

This study examined the relationships among PS, PSQI, PE, and SI in a sample of Chinese graduating-grade secondary school students. The results showed that PS was significantly and positively associated with SI, and that PSQI partially mediated this association. PE was not only associated with lower levels of SI, but also moderated the strength of the associations of PS and PSQI with SI. Supplementary machine-learning analyses indicated that PSQI-related sleep problems, PS, PE, and their interaction terms made substantial predictive contributions to SI. Further network analysis showed that subjective sleep quality, sleep latency, sleep disturbances, stress-coping burden, and PE frequency exhibited relatively high connectivity within the overall network. Overall, SI among graduating-grade secondary school students was closely related to stress experiences, sleep problems, and PE levels. School-based mental health services should therefore pay particular attention to sleep status and exercise participation among students exposed to high stress, and should integrate stress management, sleep support, and regular PE into comprehensive mental health promotion strategies.

## Data Availability

The datasets generated and/or analyzed during the current study are available from the corresponding author upon reasonable request.
